# Screening and Diagnosis of Chronic Pharyngitis Based on Deep Learning

**DOI:** 10.3390/ijerph16101688

**Published:** 2019-05-14

**Authors:** Zhichao Li, Jilin Huang, Zhiping Hu

**Affiliations:** 1 School of Political Science and Public Administration, East China University of Political Science and Law, Shanghai 201620, China; 2863@ecupl.edu.cn; 2 College of Environmental Sciences and Engineering, Peking University, Beijing 100871, China; yellowjilin@gmail.com

**Keywords:** Chronic pharyngitis, Deep learning, Convolutional neural network, Spectrogram

## Abstract

Chronic pharyngitis is a common disease, which has a long duration and a wide range of onset. It is easy to misdiagnose by mistaking it with other diseases, such as chronic tonsillitis, by using common diagnostic methods. In order to reduce costs and avoid misdiagnosis, the search for an affordable and rapid diagnostic method is becoming more and more important for chronic pharyngitis research. Speech disorder is one of the typical symptoms of patients with chronic pharyngitis. This paper introduces a convolutional neural network model for diagnosis based on the typical symptom of speech disorder. First of all, the voice data is converted into a speech spectrogram, which can better output the speech characteristic information and lay a foundation for computer diagnosis and discrimination. Second, we construct a deep convolutional neural network for the diagnosis of chronic pharyngitis through the design of the structure, the design of the network layer, and the description of the function. Finally, we perform a parameter optimization experiment on the convolutional neural network and judge the recognition efficiency of chronic pharyngitis. The results show that the convolutional neural network has a high recognition rate for patients with chronic pharyngitis and has a good diagnostic effect.

## 1. Introduction

Chronic pharyngitis is a chronic inflammation of the pharyngeal mucosa, submucosa, and lymphoid tissues. According to pathology, it is divided into simple catarrhal pharyngitis and hypertrophic pharyngitis [1]. Chronic pharyngitis is an inflammation of the upper respiratory tract, which is stubborn and difficult to cure. Symptoms of chronic pharyngitis vary from person to person. Usually, the pharynx has various discomforts, such as foreign body sensation, burning sensation, and language disorder [2]. The etiology of chronic pharyngitis is complex. Bacterial infection is the most important cause, followed by non-infectious factors, such as obstructive sleep apnea and hypopnea syndrome, and occupational exposure. The pathogenesis of chronic pharyngitis mainly includes neurophysiological mechanisms and the L-form mechanism [3].

In the United States, the number of outpatient visits for chronic pharyngitis ranges from 7,000,000 to 11,000,000 [4,5,6]. In China, the incidence of chronic pharyngitis is extremely high due to eating habits, environmental factors, and antibiotic abuse. The proportion who suffer from the illness is close to one third of the population [7]. In a statistical study of 1100 people who underwent physical examination, researchers concluded that the incidence of chronic pharyngitis in this group was as high as 78.65% [8,9]. The above data shows that chronic pharyngitis has always had a high incidence rate in countries all over the world, especially in large cities with poor air quality, which brings many troubles and burdens to the majority of patients and takes up a lot of medical resources [10].

Due to the persistent symptoms of chronic pharyngitis, the medical profession generally believes that it is necessary to detect and delay the development of the disease through some controlled drugs as soon as possible. Diseases similar to chronic pharyngitis are difficult to diagnose at present. They are easy to be misdiagnosed with some diseases such as chronic tonsillitis, chronic laryngitis, pharyngeal and laryngeal tumors, and cervical spondylosis. [11]. The current early diagnosis of chronic pharyngitis mainly relies on the doctor’s clinical experience to ask the patient’s medical history to reach a conclusion, and the positive predictive value based on the doctor’s clinical experience diagnosis is only 75% [12]. Computed tomography can improve the accuracy rate of early diagnosis of most diseases, but due to the high cost of computed tomography, it is impractical for the diagnosis of chronic pharyngitis. In order to reduce costs and avoid misdiagnosis, the search for an affordable and rapid diagnostic method is becoming more and more important for chronic pharyngitis research.

Speech disorder and audio frequency reduction are typical symptoms of chronic pharyngitis. Most patients with chronic pharyngitis have symptoms of speech disorder and audio reduction [13]. For chronic pharyngitis, it is easy to miss the best treatment time because there are no biological indicators for diagnosis in the early stage, the diagnosis cost is high, and there are limitations in the operation, so the probability of misdiagnosis is greatly increased, leading to the gradual increase of potential patients with chronic pharyngitis [14]. In recent years, the diagnosis of chronic pharyngitis based on speech disorder has gradually become a hot spot of extensive research [15]. Research shows that with the continuous progress of machine learning technology, it is feasible to diagnose chronic pharyngitis by using machine learning through speech disorder. The diagnosis based on speech disorder can transmit vocalization through a microphone and analyze the voice signal, so as to get a preliminary diagnosis for patients. Compared to other methods, this diagnostic method is simple and inexpensive.

At present, some scholars have made a preliminary analysis of other diseases using pattern recognition methods, such as Max A. Little of Oxford University, who diagnosed Parkinson’s patients through speech disorder recognition, and C. Okan Sakar of Bogazici University, who classified post-traumatic stress disorder using a support vector machine, and achieved good accuracy [16,17,18,19]. This paper attempts to introduce a method based on the deep neural network to detect speech disorders in chronic pharyngitis. A complex convolutional neural network is used for self-learning features in speech, and the features are improved by adjusting the network continuously.

## 2. Materials and Methods

### 2.1. Deep Learning Methods

At present, disease diagnosis based on speech disorder is accomplished by mapping features into vector space. In fact, this method uses pattern recognition to solve speech disorder problems. Its advantage is that it is simple to calculate and can be deduced by mathematical formulas, which is easy to popularize. However, this method has some limitations. For example, the final classification result may be unconvincing, and the result is often overfitting.

In recent years, the emerging deep learning method can effectively control the redundancy of feature extraction algorithms and improve the analysis efficiency [20]. In the feature extraction process, deep learning simulates the thinking of the human brain for self-learning, effectively avoiding problems such as redundancy of feature information and uncertainty of new features [21]. Compared with the traditional artificial neural network, deep learning is a deeper network model [22,23,24]. Common deep learning models include AutoEncoder, Deep Belief Networks, Restricted Boltzmann Machine, and Convolutional Neural Networks. As one of the common models of deep learning, convolutional neural networks are widely used in the field of image recognition and speech recognition [25,26]. In this paper, the convolutional neural network model is used to diagnose speech disorders in chronic pharyngitis.

### 2.2. Convolutional Neural Network Theory

The essence of deep learning is the extraction of features of data through nonlinear transformation of input and extraction parameters of a multi-hidden layer neural network through supervised or unsupervised training for feature extraction and classification [27]. Compared with the traditional neural network, the convolutional neural network not only increases the depth of the network model, but also performs convolution and down-sampling operations on the input based on its own network features of weight sharing and local perception, which significantly improves the operation efficiency and precision.

The traditional artificial neural network is a fully connected network. The neurons in two adjacent network layers show a multi-to-one relationship, and all the neurons in the former layer are connected with each neuron in the lower layer. In this case, if the amount of data is large, it will cause too many parameters in the training process and slow down the training speed. When the input object is a picture, the picture will be shifted, and the neural network will recognize the picture as different from the picture before the displacement because of the moving of the pixels. At the same time, due to the structural characteristics of full connection, the neural network cannot recognize the local features in the image. The convolutional neural network is improved on the basis of the neural network, and its feature weight extraction is further optimized by weight sharing.

The input of the convolutional neural network is always two-dimensional, and the pixel of the two-dimensional image is regarded as a neuron. Unlike the neural network, the local neurons are connected to the next layer of neurons instead of the full connection in the neural network. In this way, external edges and endpoints of the image can be combined, and more features can be extracted.

In the traditional artificial neural network, the neurons in the network layer are connected in a fully connected manner, and each hidden layer contains multiple features. In a convolutional neural network, the weights of neurons in each hidden layer connected to their upper layer are combined into a matrix as a convolution kernel. Using the same convolution kernel and the input image for the convolution operation, in the back propagation of error function, the weight of the convolution kernel is a process of continuous learning, that is, constantly updating the weight of the convolution kernel.

Since there is a convolution kernel in each neuron in the hidden layer, the same convolution kernel is convolved with the input image of the previous layer, and the combination of the generated feature vectors is the feature of the neurons connected to the current layer. This greatly reduces the number of parameters that need to be trained during the training process.

### 2.3. Back Propagation Algorithm

**Figure 1:**
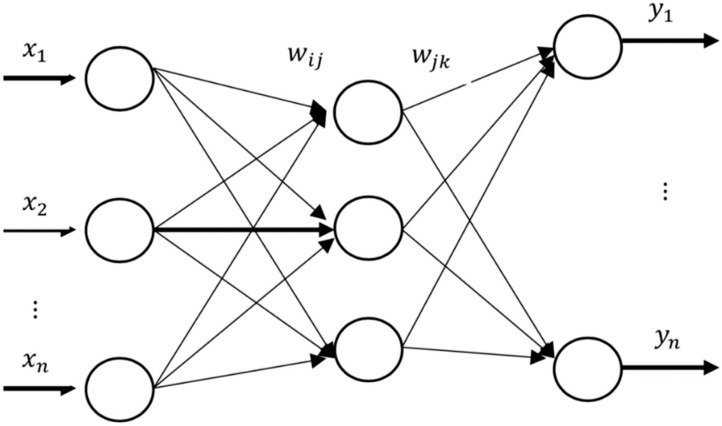
Topology of Neural Networks.

The back propagation algorithm was proposed by Paul Werbos in 1974 [28]. The back propagation algorithm is a supervised learning algorithm that requires the activation function of neurons to satisfy differentiable conditions [29]. It is generally suitable for training in the forward network. The update of network parameters is completed through the transmission of difference values. Taking the simple neural network in Figure 1 as an example, the back propagation algorithm is briefly introduced.

The input is $\vec{x}\left(x_{1},x_{2},\cdots x_{i}\right)$ and the target output is $\vec{t}\left(t_{1},t_{2},t_{3},\cdots t_{k}\right)$. Firstly, the network parameters are initialized. According to the number of input data and the number of target output vectors, the parameters of each network layer are set. The number of nodes in the input layer is *M*, the number of nodes in the final output layer is *C*, and the number of nodes in the hidden layer is *H*. The weights between the input layer and the hidden layer are initialized as ${w_{ij}}^{n}$. The weights between the output layer and the hidden layer are initialized as ${w_{jk}}^{n}$. The hidden bias unit is initialized as ${b_{k}}^{n}$. The appropriate learning rate *u* and activation function are selected. When initializing the network, the weight needs to be set to a random value with a small value.

According to input $\vec{x}$, connection weight ${w_{ij}}^{n}$, and bias unit ${b_{j}}^{n}$, the output of hidden layer ${h_{j}}^{n}$ is obtained.

(1) $${h_{j}}^{n}=f\left(\sum_{i=1}^{M}{w_{ij}}^{n}{x_{i}}^{n}-{b_{j}}^{n}\right)j=1,2,\cdots ,H$$

f is the activation function. The commonly used activation functions are the sigmoid function, tanh function, and rectified linear unit (ReLU) function. The ReLU function can not only accelerate the convergence of the stochastic gradient descent algorithm, but also reduce the training time. It has linear and unsaturated characteristics and can be activated by a simple zero threshold matrix.

According to the output of the hidden layer ${h_{j}}^{n}$ and the connection weight ${w_{jk}}^{n}$ and bias unit ${b_{k}}^{n}$, the output of the output layer ${y_{k}}^{n}$ is obtained.

(2) $${y_{k}}^{n}=\sum_{j=1}^{H}{h_{j}}^{n}{w_{jk}}^{n}-{b_{k}}^{n},k=1,2,\cdots ,C$$

The goal of the back propagation algorithm is to get the output of the neural network by forward propagation of the training samples. According to the difference between the actual output and the expected output, the back propagation process is stopped when the error function of the network reaches the minimum value, and the weight and bias unit of each neuron in each layer are updated in turn from the output layer to the hidden layer and from the hidden layer to the input layer. The target output is ${t_{k}}^{n}$, and the total error function of the training samples is obtained by forward propagation and is the total number of samples.

(3) $$E^{N}=\frac{1}{2}\sum_{n=1}^{N}\sum_{k=1}^{C}{\left({t_{k}}^{n}-{y_{k}}^{n}\right)}^{2}$$

The sensitivity δ is introduced to represent the reciprocal of the mean square error (MSE) to the bias unit, that is, the step size of the bias unit change.

(4) $$\frac{\partial E}{\partial b}=\frac{\partial E}{\partial u}\frac{\partial u}{\partial b}=\delta$$

$\frac{\partial E}{\partial b}=\frac{\partial E}{\partial u}=\delta$ is obtained from $\frac{\partial u}{\partial b}=1$, that is, the partial derivative of error function to bias unit is the same as that of error function to its input, T is the symbol of the transposed matrix, and the sensitivity of hidden layer is as follows:

(5) $$\delta^{l}={\left(W^{l+1}\right)}^{T}\delta^{l+1}\circ f\left(u^{l}\right)$$

“∘” means that each element is multiplied and the sensitivity of the output layer is:

(6) $$\delta^{L}=f^{\prime }\left(u^{L}\right)\circ \left(y^{n}-t^{n}\right)$$

Finally, we judge whether the error value $E^{N}$ of the actual output of the network and the target output satisfies a certain threshold. If it is less than this threshold, the entire training process has reached the global optimum. If the specified threshold is exceeded, back propagation continues. In the back propagation process, the error value $E^{N}$ is transmitted in the opposite direction of the forward propagation. Through the reverse transmission process of the error value $E^{N}$, the weights and bias unit of the layers are sequentially updated until the error is less than a certain threshold.

### 2.4. Topological Structure of Convolutional Neural Networks

**Figure 2:**
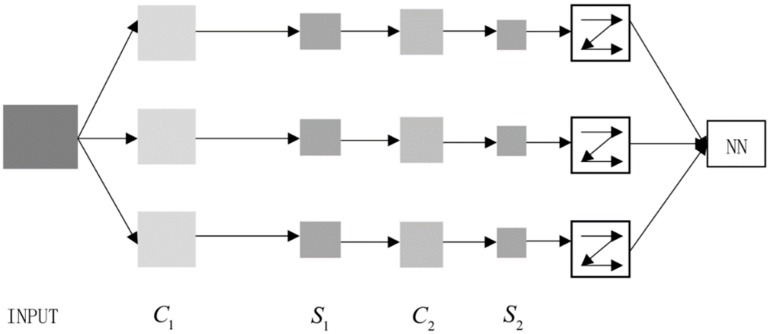
Convolutional neural network (NN) structure. C is convolution and S is down-sampling.

A convolutional neural network is a deep structure of artificial neural networks, which consists of an input layer, multiple hidden layers, and an output layer. As shown in Figure 2, there are several S (down-sampling) and C (convolution) layers in the network. The S-layer is composed of S-planes and S-planes are composed of S-elements (simple elements). The structure of layer C is the same as layer S. Layer C consists of many C planes and C elements.

The purpose of the convolution operation after the down-sampling layer is to perform secondary extraction of features. The tandem structure of the convolutional layer and the down-sampling layer enables the network to recognize different forms of input samples with higher adaptability.

Input layer: place the same size image (r×c size) in the input layer. r is the value of the row and c is the value of the column.

Convolution layer $C_{1}$: set the convolution kernel size to a×b, the step size to i, and convolute the convolution kernel with the input. The size of the output matrix is d×e. The convolved result forms the convolution layer. a,b,d,e are unknown parameters used to represent the specific value of the variable. It should be noted that: a<r,b<c.

(7) $$d=\frac{r-a+i}{i},e=\frac{c-b+i}{i}$$

The three convolution kernels convolve the input data separately and add them to the corresponding bias unit. f is the activation function, b is the corresponding bias unit, l is the number of layers, and *k* is the convolution kernel.

(8) $${x_{j}}^{l}=f\left(\sum_{i\in M_{j}}{x_{j}}^{l-1}*{k_{ij}}^{l}+{b_{j}}^{l}\right)$$

**Figure 3:**
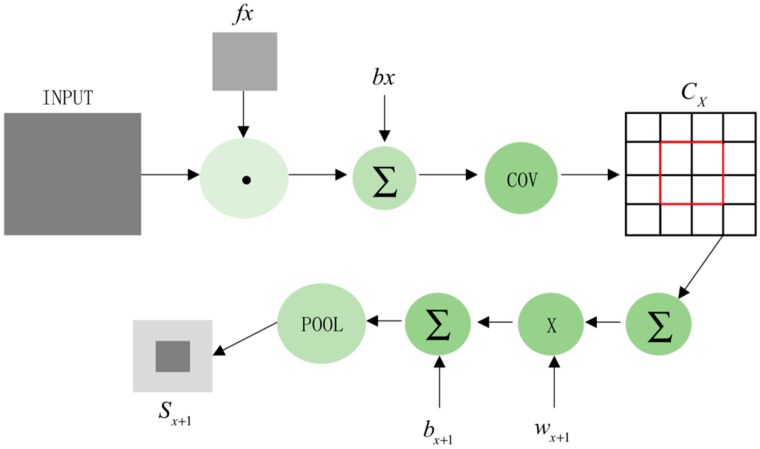
Convolution (COV) and down-sampling operations.

Down-sampling layer $S_{1}$: down-sampling the output of the $C_{1}$ layer. After weighted summation or maximization of the n×n matrix of the input characteristic graph, it multiplies the corresponding multiplicative deviation, adds a bias, and finally obtains three characteristic graphs through an activation function. The two dimensions of the feature graph are reduced to $\frac{1}{n}$ of the input graph. While increasing the training speed of the network, the original information will not be lost and the amount of data will be reduced.

(9) $${x_{j}}^{l}=f\left({\beta_{j}}^{l}down\left({x_{j}}^{l-1}\right)+{b_{j}}^{l}\right)$$

where “down” in the above formula is a down-sampling function, *b* is an additive bias unit, and β is a multiplicative bias unit. As shown in Figure 3, the steps in the figure are, respectively, convolution operation and down-sampling operation. After repeated operations, the dimension of the intermediate feature graph decreases, and the matrix of the feature graph is expanded in the last convolution. After the rasterization operation, a one-dimensional vector is generated, and a classifier with differentiable weights is added. The larger the dimension of data, the more convolution operations and down-sampling operations there are.

### 2.5. Spectrogram

In this paper, the convolutional neural network is used to represent the time–frequency graph of the speech disorder, and the network model is updated to obtain the final classification accuracy. Speech is a one-dimensional signal, which is represented by one-dimensional feature vectors in space [30]. The convolutional neural network can extract features from data by self-learning through convolution and down-sampling operations. It guarantees the appropriate learning rate and robustness of the model. The one-dimensional speech signal only represents the characteristics of speech in the time domain. As the input of the convolutional neural network, it may cause too few features due to the constraints of the time domain, which may reduce the reliability of the network and even cause the over-fitting of the network. Considering the problems that one-dimensional signals may cause, this paper tries to use two-dimensional speech signals (time domain and frequency domain) [31].

The time–frequency change of speech refers to the transformation of speech signals (one-dimensional form) into a spectrogram (two-dimensional form) by short-time Fourier transform. The window length and the number of points of the Fourier transform are very important. The parameter setting has a crucial influence on the accuracy of the convolutional neural network model. The process of the Fourier transform is as follows:

x(m) is input signal at time m, $X\left(e^{jw}\right)=\sum_{n=-\infty }^{\infty }x\left[n\right]e^{-jwn}$ is Discrete-time Fourier Transform of the forward transformation, w(n−m) is a window function (Hanmming window).

(10) $$X_{n}\left(e^{jw}\right)=\sum_{m=-\infty }^{\infty }x\left(m\right)\cdot w\left(n-m\right)e^{-jwn}$$

Due to the speech signal is too long to be processed all at once, the function of segmentation is Hanmming window. $X_{n}\left(e^{jw}\right)$ is a function of w and n, and w=2πk/N(0≤k≤N−1). The short-time Fourier transform of the speech signal is as follows:

(11) $$X_{n}\left(k\right)=X_{n}\left(e^{2\pi kj/N}\right)=\sum_{m=-\infty }^{\infty }x\left(m\right)\cdot w\left(n-m\right)\cdot e^{-2\pi kj/N}$$

The window length of the Fourier transform is w(n−m) and the number of points of the Fourier transform is N.

The language spectrogram contains a large amount of information about the speaker’s features, which breaks through the limitations of feature constraints in the audio domain and time domain, and dynamically presents the characteristics of signal spectrum changes. Therefore, in order to meet the input requirements of convolution neural network and feature extraction, this paper uses the spectrogram as the input of the convolution neural network.

In a spectrogram, the vertical axis is frequency and the horizontal axis is time. The intensity of any frequency at a given time is expressed by the gray level or tone of the corresponding points in the graph [32]. The color depth of the gray value of the pixels in the spectrograms is used to represent the energy.

**Figure 4:**
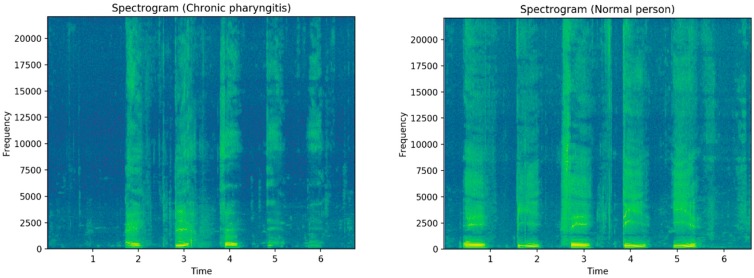
Comparison of the spectrum of patients with chronic pharyngitis and normal people.

Figure 4 shows the comparison of a speech spectrum between normal people and patients with chronic pharyngitis. We can make the following two observations: the first is a change of the format in the frequency domain when the time is constant; the second is the change in loudness in adjacent time when the frequency is constant. We can find that the frequency of the spectrum of patients with chronic pharyngitis is generally low, mainly distributed below 5000, while the frequency of normal people is higher, and the distribution is more uniform.

### 2.6. Different Deep Learning

#### 2.6.1. Recurrent Neural Networks

On the basis of Multilayer Feedforward Neural Network, Recurrent Neural Networks (RNNs) we can combine the concept of time, provide a memory function for the neural network, and make the neural network show good modeling ability on time–series data. However, the common problem caused by too many layers in the time dimension is the disappearance or explosion of the gradient. The long short-term memory neural network theory (LSTM) proposed by Hochreiter and Schmidbuber solves this problem very well [33]. The architecture of RNN analyzed in this paper is long short-term memory neural network.

**Figure 5:**
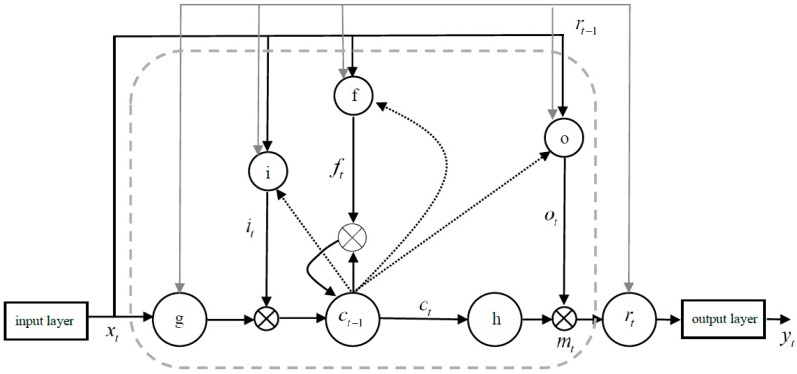
Long short-term memory neural network (LSTM).

As shown in Figure 5, i,f,o three gate structures are represented in LSTM, respectively. The activation functions of the three gates are Sigmoid. c is the memory unit of LSTM. The dot product and sum of the matrices are represented by m,g respectively. r is the loop in the time dimension. t is the current time, and t−1 is the last time at the current time. These gates make the control of LSTM more precise in the training process, but on the other hand, these gates increase the computational complexity. In order to improve the training speed without reducing the number of gates, it is necessary to linearly express the activation value.

**Figure 6:**
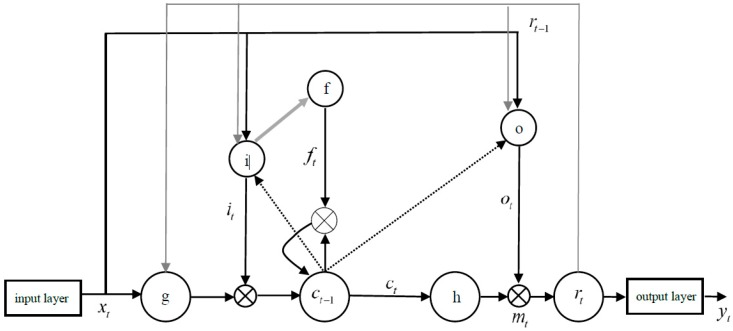
The binarized long short-term memory neural network.

In this paper, a binarization method is proposed to speed up training of the network. We assume the threshold to be 0.85 and consider the activation value greater than or equal to the threshold as 1. Otherwise, we assume the threshold to be 0, thus accelerating the training and decoding of the network. Then the activation value of the gate is binarized, and one gate is used to represent the other gate linearly. The binarized LSTM is shown in Figure 6.

In this paper, the mini-batch of gradient descent is 512, the learning rate of initial training is 0.0006, and the learning rate of end training is 0.00006. In the training process, when the loss does not decrease as the training progresses, the learning rate can be appropriately reduced.

#### 2.6.2. Deep Belief Networks

**Figure 7:**
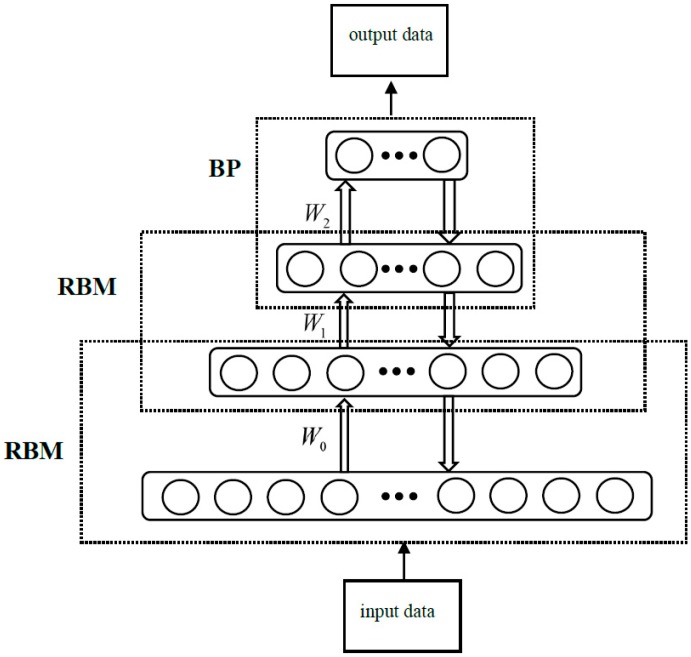
The structure of Deep Belief Networks (DBNs).

As shown in Figure 7, Deep Belief Networks (DBNs) are composed of multi-layer Restricted Boltzmann Machines and a layer of the back propagation neural network.

Multi-layered Restricted Boltzmann Machines (RBMs) use unsupervised learning methods, while BP neural networks use supervised learning methods. The operation of DBN is divided into two stages. The first stage is the pre-training phase. The output of the RBM model in the following layer is used as the input of the upper RBM. The greedy unsupervised learning algorithm is used to initialize the parameters of the entire DBN model layer by layer. The second stage is the fine-tuning stage. The entire deep-structure learning algorithm optimizes and adjusts the relevant parameters of the network space and fine-tunes the model from top to bottom. This method makes DBN perform well in semi-supervised learning tasks with insufficient training data. This training mode effectively reduces the space for parameter optimization through unsupervised training, greatly reducing the time for supervised training [34].

In this paper, the number of layers of DBN is 5, the number of neural units per layer is reduced layer by layer, and the order of reduction is 32–20–10–5–2. The momentum of unsupervised learning in DBN is 0.5, the learning rate is 0.5, and the number of iterations is 100.

#### 2.6.3. Convolutional Neural Network (SqueezeNet)

The convolutional neural network architecture used in this paper is AlexNet, which requires high computing resources, storage space, and power consumption. The SqueezeNet architecture has been widely used due to its good prediction accuracy and few model parameters. SqueezeNet is an architecture of the convolutional neural network (CNN), but unlike the general CNN-like architecture, SqueezeNet minimizes model depth and parameters while maintaining a certain model prediction accuracy [35]. To achieve this goal, SqueezeNet adopts the following three strategies: ① The size of most convolution kernels in the network is 1×1. ② The number of input channels of the remaining convolution kernel (3×3) is reduced by squeezing the convolution layer. ③ The delayed down sampling ensures that the convolution layer has a larger activation map.

In this paper, the optimizer selected for the training is Nesterov, the weight decay factor is 5e–4, the momentum is 0.9, the initial learning rate is 0.01, and the learning rate reduction strategy is polynomial attenuation.

## 3. Results

### 3.1. Data Preprocessing

In this paper, a convolutional neural network model was used to diagnose chronic pharyngitis based on clinical samples. The experiment was based on the pretreatment of speech data. The data used in this experiment were the voices of patients with chronic pharyngitis and the voices of healthy people. Acquisition of good voice signals was the base data of the experiment.

In this paper, 730 speech data of chronic pharyngitis were selected. The other data source was the voice signals of healthy people, which contains 500 data. The length of each speech segment was 6 s. When choosing phonetic elements, five vowels in English international phonetic symbols were selected as speech signals. Since similar pronunciation can be found in all kinds of languages in the world, the selection of pronunciation has wide applicability.

When collecting voice data, it is necessary to ensure the quietness of the surroundings and the integrity of the voice. The collected speech data were transformed into spectrograms under specified conditions, which were used as inputs of the convolutional neural network model, and were trained and tested to get the correct rate of diagnosis.

### 3.2. Experimentation

In this experiment, the experimental results were compared by setting different parameters and different conditions, and the optimal parameters of the network model were obtained, and thus the final recognition rate was obtained. On the basis of this experiment, the data set was trained and tested on DBN and RNN, and the recognition rate was obtained. The performance of convolutional neural networks and other deep learning networks were compared from the final recognition rate.

For the purpose of the diagnosis of chronic pharyngitis, the speech of all patients with chronic pharyngitis and normal people were expressed in time–frequency and input into the convolutional neural network constructed in this paper, and the final results were output in turn in the console.

This experiment used the recognition rate of each spectrum as the evaluation index of the object. When the probability of the output of one category was greater than that of the other category and was consistent with the label, it was correct to judge whether the object had chronic pharyngitis. Conversely, the model misjudged whether the patient had chronic pharyngitis. By inputting all the test sets into the network, the correct judgment ratio was obtained, which was used as the evaluation index of the performance of the convolutional neural network. The recognition rate was compared with other deep neural network algorithms to judge the performance of the convolution neural network.

The experiment was divided into two steps. Firstly, the parameters of the convolution neural network were selected to ensure the maximum recognition rate. The main parameters affecting the convolutional neural network were learning rate, number of output nodes, and momentum value. The first step was to make a comparative experiment with different learning rates, then compare the number of nodes in different middle tiers, and then select the appropriate momentum value to carry out the experiment. The optimal parameter values of each scheme were selected as default parameters of the convolution neural network. The second step was to compare the convolutional neural network with the deep belief network (DBN) and recurrent neural networks (RNN), and to illustrate the advantages and disadvantages of the convolutional neural network in the diagnosis and recognition of chronic pharyngitis disease by comparing the final results.

The speech sampling frequency of the spectrogram was 22050 Hz, monophonic, wav format. There were 1230 training samples, 730 diseased samples, and 500 normal samples. There were 100 test samples, of which 70 were chronic pharyngitis samples and 30 were normal samples. The set parameters were as follows: the number of points of the Fourier transform was 1024, the overlap length was 512, and the window length of the Fourier transform was 1024.

### 3.3. Learning Rate Comparison Experiment

The learning rate has a significant impact on the final classification effect. When the learning rate is too small, the algorithm will easily converge during training. If the learning rate is too large, it will speed up the learning process, causing the accuracy curve to oscillate or diverge. This experiment used five different learning rates for comparative testing, which were 0.5, 0.1, 0.01, 0.001, and 0.0001. In this experiment, except for the learning rate, the other parameters were the same. Table 1 shows the results of comparative experiments with different learning rates.

From the comparison of the experimental results in Table 1, it can be concluded that the difference in learning rate led to different recognition rates. According to the above experimental results, it can be observed that as the number of iterations increased, the learning ability of the network tended to be stable, and the recognition rate gradually converged. When the learning rate was 0.01, the recognition rate was the highest, so the learning rate of the convolutional neural network was 0.01.

### 3.4. Output Node Experiment

In convolutional neural networks, the number of output nodes has an important impact on the performance of the whole network. In the diagnosis of chronic pharyngitis, it is necessary to verify the impact of the number of different output nodes on network performance and the optimal solution. This was done by setting the number of output nodes to 1024, 2048, and 4096, respectively, and observing the change of the recognition rate of the convolutional neural network under the number of different output nodes.

**Figure 8:**
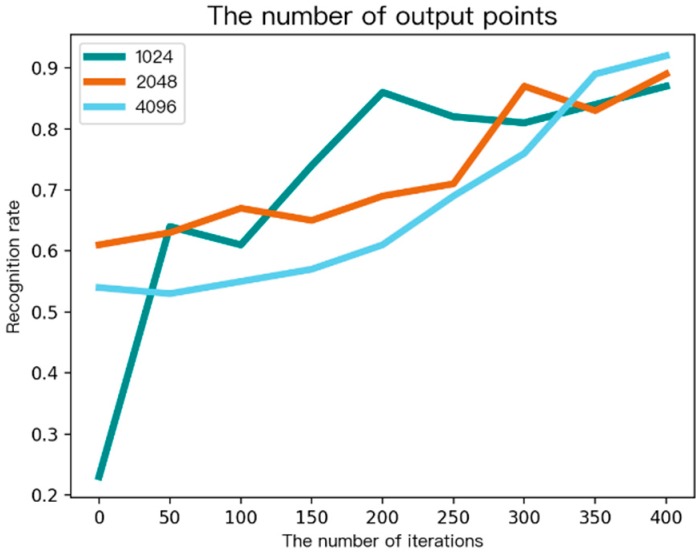
Recognition rate under different output nodes.

As shown in Figure 8, the recognition rates of the three experiments were almost the same, and the final accuracy reached about 90%. It can be seen that in the process of designing the convolutional neural network, it was necessary to set the number of output nodes of the fully connected layer according to the number of input data samples, and the number of output nodes of the fully connected layer was not a key factor directly affecting the recognition rate.

### 3.5. Momentum Experiment

In the early stage of the network training process, the initial operation of the weights of the network are carried out. Generally, it follows some distribution, such as Gauss distribution. Initialization of network weights has a great impact on the final classification performance of the network, that is, if the network weights are set properly, the convergence speed of loss function in the process of network training will be accelerated, and the optimal value can be achieved as soon as possible. If the initialization weights are set inappropriately—too large or too small—the loss function of the network will fall into the local minimum and not reach the global optimal value.

The setting of momentum can solve this problem to some extent. The concept of momentum value is put forward according to the transformation relationship between potential energy and kinetic energy in physics. The greater the value of momentum, the greater the value of potential energy. In the convolutional neural network, choosing the appropriate momentum value enables the loss function of the network to enter the global concave region without being bound in the local concave region. Table 2 shows the recognition rate comparison with different momentum values.

It can be seen from Table 2 that when the momentum value is 0.95, the network’s recognition rate of speech spectrogram reaches an optimal effect. For data sets, slightly higher or lower momentum values have a certain impact on the accuracy of the network’s final recognition of speech spectrogram.

### 3.6. Comparison of Different Deep Learning Effects

After determining the parameters of the convolutional neural network model, the prepared data set was used for model training and testing. The higher the recognition rate of the model, the better the diagnosis effect of the model on chronic pharyngitis. The lower the recognition rate, the greater the defect of the model.

At the same time, in order to more intuitively illustrate the advantages of the convolutional neural network model in the diagnosis of chronic pharyngitis, we also compared the diagnostic results of using Deep Belief Networks (DBN), Recurrent Neural Networks (RNN), and Convolutional neural network (SqueezeNet) to process the same data set, and used the results to judge the advantages and disadvantages of the diagnostic model of chronic pharyngitis.

Table 3 shows the performance comparison of different algorithms for the chronic pharyngitis data set.

As seen in Table 3, the diagnosis effect of the convolutional neural network was higher than DBN, RNN, and CNN (SqueezeNet). Since the DBN architecture does not take into account the two-dimensional structure of the image (one-dimensional vectorization of the image matrix), the recognition rate for the image was low [36].Therefore, it can be concluded that, based on the experimental data set, DBN and RNN have poor applicability, and the convolutional neural network has a higher recognition rate [37]. Based on the experimental results of the two steps of selecting the parameters of the convolutional neural network and comparing the recognition rates of different models, it can be seen that the recognition rate of the convolutional neural network constructed in this paper based on the diagnosis of speech disorders in chronic pharyngitis was higher than DBN, RNN, and CNN (SqueezeNet). This method reduced the redundancy of the features and visualized the features while ensuring the final diagnostic rate.

## 4. Conclusions

The work of this paper is mainly divided into three parts: ① The conversion of speech time–frequency. Based on the limitations of signal feature extraction in the time domain, a method of feature extraction by converting speech into spectrum is introduced. We try to classify chronic pharyngitis speech data as an input of the deep learning algorithm. In view of the advantages of the convolutional neural network in feature extraction from multi-dimensional images, a method based on the convolutional neural network is introduced to diagnose speech disorders in chronic pharyngitis. In the early stage of data processing, speech signals are converted into spectrograms with both time-domain and frequency-domain characteristics. Compared with the previous feature extraction in single time or frequency domains, it is more conducive to feature self-learning and new feature discovery. ② The construction of the convolution neural network. Firstly, the algorithm of the convolution neural network is introduced. In the training process, the convolution kernel and bias unit are updated by back propagation. Then, a convolution neural network suitable for the chronic pharyngitis data set is constructed. By designing the structure of the convolution layer and the down-sampling layer, the number of output nodes of the fully connected neural network is reasonably arranged, and the data transfer process is explained. ③ Parameter selection experiment and diagnostic performance test. The convolution neural network is used to carry out different comparative experiments on its parameters. By choosing appropriate parameters, the diagnostic performance of the model for data sets can be optimized, and the final diagnostic rate is 81.2%. Compared with other algorithms, the results show that the convolutional neural network designed in this paper can effectively diagnose chronic pharyngitis through speech disorders.

In this paper, a multi-contrast experiment of chronic pharyngitis speech data sets is performed by convolutional neural network, which can diagnose patients with chronic pharyngitis to a certain extent. However, in view of the small number of sample sets used in this experiment, it may affect the final diagnosis rate, which is also one of the directions for future research.

## Figures and Tables

**Table 1:** Comparative experiment of different learning rates.

Learning Rate/Number of Iterations	50	100	150	200	250
0.0001	63.72%	65.96%	72.19%	72.41%	72.86%
0.001	68.18%	69.44%	69.79%	69.79%	69.79%
0.01	72.59%	82.57%	82.73%	82.91%	82.94%
0.1	37.21%	45.63%	48.39%	51.06%	51.06%
0.5	21.54%	22.82%	23.77%	23.77%	23.76%

**Table 2:** Recognition rate under different momentum values.

Momentum Value	Recognition Rate
0.55	0.59
0.75	0.67
0.95	0.89
1.35	0.73

**Table 3:** Comparison of different algorithms for chronic pharyngitis.

Algorithm	Recognition Rate
DBN	69.3%
RNN	74.7%
CNN (AlexNet)	81.2%
CNN (SqueezeNet)	80.7%
